# Impact of Telemonitoring on Hospitalization and Mortality Among Patients With Heart Failure: A Systematic Review of Randomized Controlled Trials

**DOI:** 10.7759/cureus.110378

**Published:** 2026-06-06

**Authors:** Neiloofar Mohmand

**Affiliations:** 1 Internal Medicine, Kabul University of Medical Sciences "Abu Ali Ibn Sina", Kabul, AFG

**Keywords:** heart failure, hospitalization, mortality, remote patient monitoring, telemonitoring

## Abstract

Heart failure (HF) is a chronic condition associated with high rates of hospitalization and mortality. Telemonitoring has emerged as a potential strategy to improve patient outcomes; however, its effectiveness remains uncertain. This systematic review evaluated randomized controlled trials (RCTs) comparing telemonitoring interventions with usual care in patients with HF. A literature search was conducted using PubMed and Google Scholar to identify relevant studies published between January 2010 and December 2025. The final literature search was performed in January 2026. Eligible studies included RCTs reporting hospitalization and/or mortality outcomes associated with telemonitoring interventions. A total of 12 RCTs were included. Telemonitoring interventions demonstrated substantial heterogeneity and included remote patient monitoring systems, implantable cardiac device-based monitoring, telerehabilitation, and remote-guided therapy optimization. Several studies demonstrated reduced hospitalization compared with usual care; however, most studies reported neutral hospitalization outcomes. Regarding mortality, only a limited number of studies reported benefit, whereas most studies demonstrated no clear difference compared with usual care. Overall, telemonitoring may provide supportive benefit in selected patients with HF, although findings remain heterogeneous and predominantly neutral, particularly regarding mortality outcomes. Further well-designed studies with standardized intervention strategies are needed to better define the patient populations most likely to benefit.

## Introduction and background

Heart failure (HF) is a major global health problem associated with substantial morbidity, mortality, recurrent hospitalizations, and healthcare costs. More than 64 million people worldwide are affected by HF, and the condition remains one of the leading causes of hospitalization among older adults. Despite advances in guideline-directed medical therapy, HF continues to carry a poor long-term prognosis and significantly impacts quality of life.

Telemonitoring has emerged as a potential strategy to improve HF management through early detection of clinical deterioration, remote symptom monitoring, and optimization of medical therapy. Telemonitoring interventions in HF include several mechanistically distinct approaches such as non-invasive home remote patient monitoring, implantable cardiac device-based remote monitoring, structured telephone support, telerehabilitation programs, and remote patient management-guided therapy optimization. These interventions aim to reduce hospital admissions, improve self-management, and potentially reduce mortality.

Several randomized controlled trials (RCTs) have evaluated telemonitoring interventions in patients with HF, although findings remain inconsistent. While some studies demonstrated reduced hospitalization and improved clinical outcomes, others showed no clear benefit compared with usual care. Differences in patient populations, intervention types, duration of follow-up, and outcome definitions may partially explain these conflicting findings.

Previous systematic reviews have suggested that telemonitoring interventions may reduce HF-related hospitalization; however, heterogeneity among interventions and evolving telemonitoring technologies have limited definitive conclusions. More recent clinical guidance has recognized that selected telemonitoring approaches may provide benefit in carefully selected patients with HF; however, recommendations remain variable depending on intervention type and supporting evidence. Contemporary HF guidelines increasingly recognize the role of digital health technologies and selected telemonitoring strategies as supportive tools in the management of patients with HF.

Therefore, this systematic review aimed to evaluate RCTs assessing the effects of telemonitoring interventions compared with usual care on hospitalization and mortality outcomes in patients with HF.

## Review

Methods

Search Strategy

A systematic literature search was conducted using PubMed and Google Scholar to identify relevant studies published between January 2010 and December 2025. The final literature search was performed in January 2026. Keywords and search terms included “heart failure,” “telemonitoring,” “remote monitoring,” “telemedicine,” “hospitalization,” and “mortality.” Boolean operators (AND/OR) were used to combine search terms appropriately across databases. Additional manual screening of references from relevant articles was also performed to identify potentially eligible studies. This review was conducted in accordance with PRISMA 2020 recommendations. No formal review protocol was registered.

Inclusion and Exclusion Criteria

Studies were included if they were RCTs involving adult patients diagnosed with HF and evaluated for telemonitoring or remote patient monitoring interventions compared with usual care. Eligible studies were required to report outcomes related to hospitalization and/or mortality. Various telemonitoring approaches were included, such as remote patient monitoring systems, implantable cardiac device monitoring, telerehabilitation programs, structured telephone support, and remote-guided therapy optimization.

Studies were excluded if they were observational studies, reviews, editorials, case reports, conference abstracts, protocol-only publications, or studies involving non-HF populations. Studies that did not report hospitalization or mortality outcomes were also excluded.

Study Selection

Studies identified through database searching were screened based on titles and abstracts. Full-text articles were subsequently assessed for eligibility according to predefined inclusion and exclusion criteria. Due to the single-author nature of this review, study screening and selection were performed by one reviewer.

Data Extraction

Data extracted from the included studies included study characteristics, sample size, patient population, intervention type, hospitalization outcomes, and mortality outcomes. Data extraction was performed manually by the author using a structured approach.

Risk of Bias Assessment

Risk of bias assessment was performed using the Cochrane Risk of Bias (RoB 2) tool for RCTs. The following domains were evaluated: randomization process, deviations from intended interventions, missing outcome data, measurement of outcomes, and selection of reported results. Each study was categorized as having low risk, some concerns, or high risk of bias. Due to the single-author design of this review, independent duplicate assessment was not performed, and this was considered a limitation of the study.

Synthesis Methods

Because of substantial heterogeneity among telemonitoring interventions, patient populations, and outcome definitions across included studies, a formal quantitative meta-analysis was not performed. Instead, findings were synthesized narratively in accordance with the principles of the Synthesis Without Meta-analysis (SWiM) reporting approach.

Results

**Figure 1:**
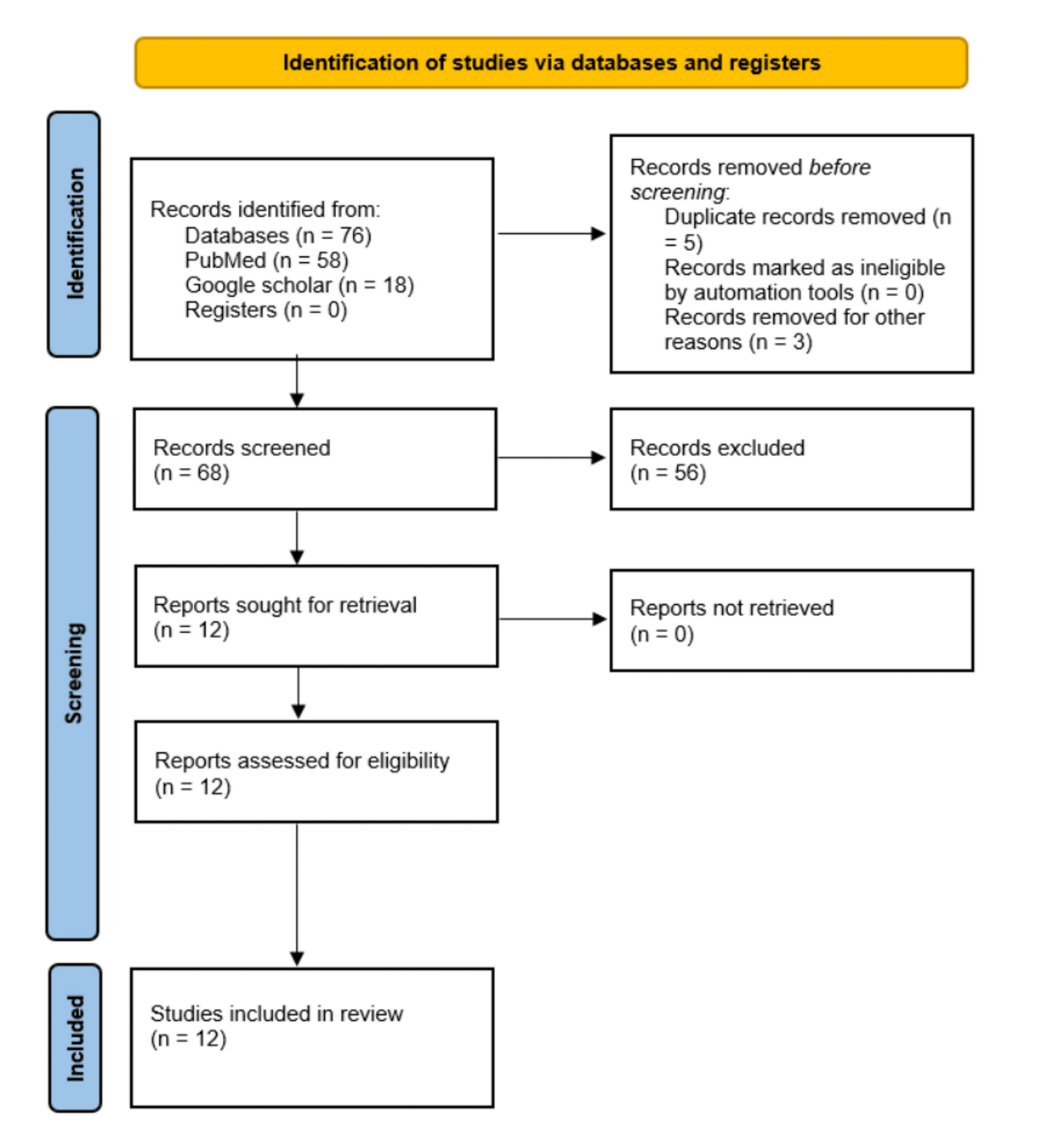
PRISMA 2020 flow diagram of study selection process

A total of 12 RCTs were included, involving patients from multiple countries and healthcare settings. Figure 1 presents the PRISMA 2020 flow diagram of the study selection process.

The characteristics of the included studies are summarized in Table 1.

**Table 1:** Characteristics of included randomized controlled trials evaluating telemonitoring in heart failure AMULET: Ambulatory Care in Heart Failure Supported by Telemedicine; CIED: cardiac implantable electronic device; ICD: implantable cardioverter-defibrillator; RPM: remote patient monitoring

Author	Year	Country	Sample Size	Intervention	Hospitalization	Mortality
Szalewska et al. [1]	2021	Poland	850	Telemonitoring	No difference	No difference
Chiu et al. [2]	2022	England	595	ICD-based monitoring	No difference	No difference
Ribeiro et al. [3]	2025	Brazil	127	Telemedicine	Reduced	Not reported
Ong et al. [4]	2016	USA	1437	Telemonitoring	No difference	No difference
Piotrowicz et al. [5]	2020	Poland	850	Telerehabilitation	No difference	No difference
Krzesiński et al. [6]	2022	Poland	296	AMULET telecare model	Reduced	Reduced
Boriani et al. [7]	2017	Spain	865	Remote monitoring	No difference	No difference
Yun et al. [8]	2025	Spain	506	Remote patient management	Reduced	Reduced
Frederix et al. [9]	2019	Belgium	142	Telemonitoring	No difference	No difference
Brahmbhatt et al. [10]	2024	USA	108	RPM-guided therapy	Reduced	Not reported
Koehler et al. [11]	2018	Germany	1571	Telemedical management	Reduced	Reduced
Hindricks et al. [12]	2014	Germany	664	CIED-based remote monitoring	Reduced	Reduced

The risk of bias assessment of the included studies is presented in Table 2.

**Table 2:** Risk of bias assessment of included randomized controlled trials

Study	Randomization	Deviations	Missing Data	Outcome Measurement	Overall
Szalewska et al. [1]	Low	Low	Some concerns	Low	Low
Chiu et al. [2]	Low	Low	Low	Low	Low
Ribeiro et al. [3]	Some concerns	Low	Low	Low	Some concerns
Ong et al. [4]	Low	Low	Some concerns	Low	Low
Piotrowicz et al. [5]	Low	Low	Some concerns	Low	Low
Krzesiński et al. [6]	Low	Low	Low	Low	Low
Boriani et al. [7]	Low	Low	Some concerns	Low	Low
Yun et al. [8]	Low	Low	Low	Low	Low
Frederix et al. [9]	Low	Low	Low	Low	Low
Brahmbhatt et al. [10]	Low	Some concerns	Low	Low	Some concerns
Koehler et al. [11]	Low	Low	Low	Low	Low
Hindricks et al. [12]	Low	Low	Some concerns	Low	Low

The included telemonitoring interventions demonstrated substantial heterogeneity and included non-invasive remote patient monitoring systems, implantable cardiac device-based monitoring, telerehabilitation programs, structured telephone support, and remote-guided therapy optimization. Because of differences in intervention design, patient populations, and reported outcomes, direct comparison across studies was limited.

Several included studies demonstrated reduced hospitalization rates associated with telemonitoring interventions [6,8,10], although the majority of trials reported neutral findings compared with usual care. Regarding mortality, only a limited number of studies showed a reduction associated with telemonitoring, whereas most studies reported no clear difference between telemonitoring and usual care.

The overall risk of bias among the included studies was generally low to moderate across most domains. Most studies demonstrated low risk of bias in the randomization process and outcome measurement. However, some studies had concerns related to missing outcome data and deviations from intended interventions. Overall, the quality of the included RCTs was considered acceptable.

Discussion

Telemonitoring has emerged as a potentially valuable strategy for improving the management of patients with HF by enabling early detection of clinical deterioration and timely clinical intervention. Previous systematic reviews have suggested potential benefits of telemonitoring in HF; however, variability in intervention design, patient populations, and outcome measures continues to limit firm conclusions [13].

Several RCTs demonstrated reductions in hospitalization rates associated with selected telemonitoring interventions. These benefits may be related to improved symptom monitoring, earlier recognition of worsening HF, enhanced patient engagement, and more efficient clinical decision-making.

However, the overall findings across studies remain heterogeneous and predominantly neutral, particularly regarding mortality outcomes. While some trials reported reductions in mortality, the majority found no clear difference between telemonitoring and standard care [2,4,5,7,9]. Differences in patient populations, intervention design, duration of follow-up, healthcare systems, and outcome definitions may partially explain these inconsistent findings.

Importantly, telemonitoring interventions differed substantially among included studies. Some trials evaluated structured telephone support or non-invasive home remote patient monitoring systems, whereas others focused on implantable cardiac device-based monitoring, telerehabilitation models, or remote-guided therapy optimization. These mechanistically distinct interventions likely have variable clinical effectiveness, which limits direct comparison across studies.

Telemonitoring strategies involving active therapy optimization, multidisciplinary management, and structured clinical follow-up appeared more likely to demonstrate favorable outcomes than passive monitoring approaches. Differences in intervention intensity, patient engagement, and responsiveness to clinical deterioration may partially explain the variability in outcomes observed across studies.

Recent clinical guidance has recognized that selected telemonitoring approaches may provide benefit in carefully selected patients with HF; however, recommendations remain variable depending on intervention type and supporting evidence. Contemporary HF guidelines increasingly recognize the role of digital health technologies and selected telemonitoring strategies as supportive tools in the management of patients with HF.

The present review adds updated evidence from more recent RCTs and highlights the continued heterogeneity and inconsistency of findings across evolving telemonitoring strategies. In addition, the review emphasizes the importance of considering telemonitoring as a heterogeneous intervention category rather than a single uniform treatment approach.

This review has several limitations. First, the literature search was limited to PubMed and Google Scholar, which may have resulted in the omission of potentially relevant studies indexed in other databases and may have affected the overall comprehensiveness of the review. Second, study screening, data extraction, and risk-of-bias assessment were performed by a single reviewer without independent duplicate assessment. Third, substantial heterogeneity among interventions, patient populations, and outcome reporting limited direct comparison across studies and precluded formal quantitative meta-analysis. Additionally, no International Prospective Register of Systematic Reviews (PROSPERO) registration or Grading of Recommendations Assessment, Development and Evaluation (GRADE) certainty assessment was performed.

Overall, telemonitoring may help reduce hospitalization rates in selected patients with HF; however, its effects on mortality and long-term clinical outcomes remain uncertain because of substantial heterogeneity among interventions and study populations. Further high-quality RCTs with standardized telemonitoring strategies are needed to better define the patient populations most likely to benefit.

## Conclusions

Telemonitoring may provide supportive benefits in selected patients with HF, particularly in reducing hospitalization rates; however, evidence regarding mortality benefit remains inconsistent. The overall findings of this review were heterogeneous because of differences in intervention design, patient populations, and outcome reporting across included studies. These findings suggest that the clinical effectiveness of telemonitoring may depend on patient selection and the specific telemonitoring strategy applied. Further high-quality studies with standardized intervention protocols and longer follow-up periods are needed to better define the patient populations most likely to benefit.
